# Hepatic Steatosis Index and the Risk of Type 2 Diabetes Mellitus in China: Insights from a General Population-Based Cohort Study

**DOI:** 10.1155/2022/3150380

**Published:** 2022-08-03

**Authors:** Xintian Cai, Jing Gao, Shasha Liu, Mengru Wang, Junli Hu, Jing Hong, Qing Zhu, Guzailinuer Tuerxun, Yujie Dang, Nanfang Li

**Affiliations:** ^1^Hypertension Center of People's Hospital of Xinjiang Uygur Autonomous Region, Xinjiang Hypertension Institute, National Health Committee Key Laboratory of Hypertension Clinical Research, Key Laboratory of Xinjiang Uygur Autonomous Region, Xinjiang Clinical Medical Research Center for Hypertension Diseases, Urumqi, Xinjiang, China; ^2^Research and Education Center of Xinjiang Uygur Autonomous Region People's Hospital, Urumqi, Xinjiang, China

## Abstract

**Purpose:**

In the Chinese population, we looked at the relationship between the hepatic steatosis index (HSI) and the risk of type 2 diabetes mellitus (T2DM).

**Methods:**

To evaluate the association between HSI and the risk of T2DM, Cox regression models were employed. Hazard ratios (HR) and 95 percent confidence intervals (CI) were computed. A stratified analysis with interaction testing was also carried out. Additionally, we evaluated the incremental predictive value of the HSI over the established risk factors using the C-statistic, the IDI, and the NRI.

**Results:**

During a median follow-up period of 2.97 years, 433 (1.97%) participants developed new-onset T2DM. The smoothing curve fit plot showed a positive correlation between HSI and the risk of T2DM. After adjusting for all noncollinear variables, the risk of T2DM increased by 62% for every 1 standard deviation (SD) increase in HSI. Subgroup analysis indicated that higher HSI levels were associated with a higher risk of T2DM in those aged < 40 years. The addition of HSI enhanced the reclassification and discrimination of established risk factors, with an IDI of 0.027 and an NRI of 0.348 (both *P* < 0.001).

**Conclusion:**

Our findings suggest that an elevated HSI is substantially associated with a greater risk of T2DM in the Chinese population. HSI has the potential to be an available and supplementary monitoring method for the management of T2DM risk stratification in the Chinese population.

## 1. Introduction

The burden of diabetes is rapidly increasing due to China's economic growth, urbanization, and aging population [1–3]. The most recent publications in China show that the country's overall prevalence of diabetes has increased to 12.8% [2]. By 2045, an estimated 693 million people in China will have diabetes [1]. Type 2 diabetes mellitus (T2DM) represents nearly ninety percent of all diabetes cases and can damage many organs and physiological systems, leading to a variety of conditions that can affect an individual's quality of life and even premature death [4–6]. It is therefore critical to identify people in the general population who are at risk for T2DM so that appropriate interventions, such as dietary advice and exercise encouragement, can be implemented at an early stage [7, 8].

Currently, the most common cause of liver disease worldwide is nonalcoholic fatty liver disease (NAFLD) [9, 10]. The development of NAFLD is strongly related to adverse lifestyle choices, insulin resistance, metabolic syndrome, and visceral obesity [11–13]. NAFLD is a robust independent risk factor for T2DM, according to an increasing body of research [14, 15]. Liver biopsy is the gold standard for NAFLD diagnosis and NAFLD severity assessment [16]. Nevertheless, for economic, practical, and safety reasons, it is not feasible to perform this operation on each patient with suspected NAFLD. To diagnose NAFLD early and to reduce the cost of screening, common clinical laboratory indicators have been widely applied to construct NAFLD-related risk scoring systems [3, 17–22]. Among these, the hepatic steatosis index (HSI) has been widely used in healthcare. Specifically, HSI is a validated risk classification scheme based on the alanine aminotransferase (ALT)/aspartate transaminase (AST) ratio, gender, and routine measurements of body mass index (BMI) [17]. HSI has been reported to be a good indicator to identify the presence or absence of hepatic steatosis [23]. Moreover, a significant correlation between HSI and fatty liver grade measured by ultrasonography has been demonstrated, a finding that suggests that HSI may reflect not only the presence but also the severity of NAFLD [23, 24]. Furthermore, similar to NAFLD, HSI is closely associated with insulin resistance and metabolic syndrome, suggesting that it may be utilized to predict the risk of T2DM in the general population [18, 25–27]. The research by Sviklāne et al. showed that HSI may be a surrogate indicator of liver fat content and metabolic syndrome in patients with type 1 diabetes mellitus [19]. Data from Wang et al. indicated that HSI is independently associated with carotid atherosclerosis in T2DM and is possibly a simple and valuable marker to evaluate the progress of macrovascular complications of diabetes mellitus [17]. More importantly, the study by Song et al. confirmed that higher HSI in early gestation was independently associated with a higher risk of gestational diabetes mellitus [28]. This cohort study presented that HSI in the first trimester may be used to predict the risk of gestational diabetes mellitus in Chinese pregnant women.

To our knowledge, no research has investigated the possible association between HSI and the risk of T2DM. Therefore, we designed the present study to investigate the association between HSI and the risk of T2DM in a Chinese population.

## 2. Material and Methods

### 2.1. Data Source

The data come from the Dryad digital repository (10.5061/dryad.ft8750v). The site allows others to gain access to the raw data for free. In the source text, the owners state that they have relinquished the relevant proprietary rights to this dataset [29]. Therefore, the database is available for secondary analysis without infringement of the owners' copyright.

### 2.2. Study Population

**Figure 1:**
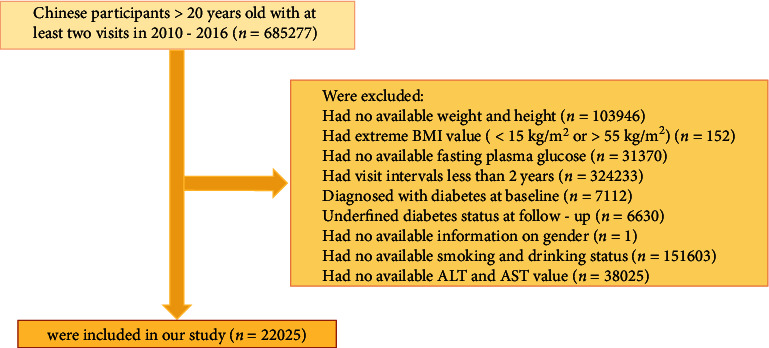
Participant flow diagram.

This study is a post hoc analysis of a cohort study conducted by the Rich Health Care Group in China; the design of which was described in detail in previous studies [29]. Briefly, the study recruited 685277 adult subjects (aged ≥ 20 years) who participated in health screenings between 2011 and 2016. The detailed selection process of participants is depicted in Figure 1. Subjects meeting the following criteria were excluded from this study: (1) no available weight and height (*n* = 103946); (2) presence of extreme BMI (*n* = 152); (3) no fasting plasma glucose (*n* = 31370); (4) visit interval less < two years (*n* = 324233); (5) diagnosed with diabetes at baseline (*n* = 7112); (6) diabetes status not determined at follow-up (*n* = 6630); (7) no available gender information (*n* = 1); (8) no available smoking and alcohol consumption status (*n* = 151603); (9) no available ALT and AST values (*n* = 38025). A total of 22,025 subjects were eventually enrolled.

### 2.3. Ethical Approval

The original clinical dataset was provided by Chen et al. [29]. No reapplication was required for this study as the study ethics had been approved in a previous study [29]. The data was anonymized and therefore did not require informed consent.

### 2.4. Data Collection

All subjects were requested to complete a standardized questionnaire containing age, family history of diabetes, gender, and smoking/alcohol consumption status. Height and weight, as well as blood pressure, were measured according to standard guidelines. Fasting venous blood samples were collected at each visit after a minimum fast of 10 h. Serum triglyceride (TG), low-density lipoprotein cholesterol (LDL-C), total cholesterol (TC), high-density lipoprotein cholesterol (HDL-C), blood urea nitrogen (BUN), ALT, serum creatinine (Scr), AST, and fasting plasma glucose (FPG) were measured by an automated analyzer (Beckman 5800). HSI was calculated as follows: HSI = 8 × (ALT/AST ratio) + BMI (+2, if female) [23].

### 2.5. Ascertainment of Incident T2DM

A diagnosis of T2DM was defined as FPG > 7.00 mmol/L and/or self-reported T2DM during follow-up. Patients were censored at the date of diagnosis of T2DM or at the last visit, whichever came first.

### 2.6. Missing Data Treatment

Missing data is an unavoidable feature of observational studies, with missing data accounting for 4.68% of all covariates in this dataset (Supplementary Table 1). To minimize bias due to missing covariates, the missing data in this study were filled using multiple interpolations, and five imputations were established. As a sensitivity analysis, this study also compared whether the imputation data differed significantly from the raw data (Supplementary Table 2). The outcomes revealed that the imputation data was not significantly different from the raw data. Therefore, the primary results of all the analyses in this paper are based on the raw data.

### 2.7. Statistical Analysis

Cox regression models were performed to estimate the relationship between HSI and the risk of T2DM, and hazard ratios (HR) and 95% confidence intervals (CI) were calculated. The collinearity among covariates was assessed by calculating VIF of covariates before building the model (Supplementary Table 3) [30]. Kaplan-Meier survival curves were then employed to show the risk of T2DM for each HSI quintile, and compliance with the proportional risk assumptions used to build the Cox model was ascertained by looking at the Kaplan-Meier curves corresponding to the HSI quintile. Following the above premise, we implemented a model adjustment strategy concerning the statement of STROBE [31]. To validate the robustness of results derived from the primary analyses, sensitivity analyses were conducted. Furthermore, we performed a stratified analysis with interaction tests. Detailed statistical methods are described in Supplementary Methods.

Statistical analyses were performed using R software, version 4.0.1.

## 3. Results

### 3.1. Characteristics of Study Participants

Of the 685277 participants enrolled in the former research, 22025 met the current inclusion criteria (Figure 1). The mean age at baseline was 41.54 ± 12.35 years, with slightly more male participants than female participants (66.07% vs. 33.93%). Baseline characteristics by HSI quintiles are summarized in Table 1.

### 3.2. Participant Follow-Up Results

**Figure 2:**
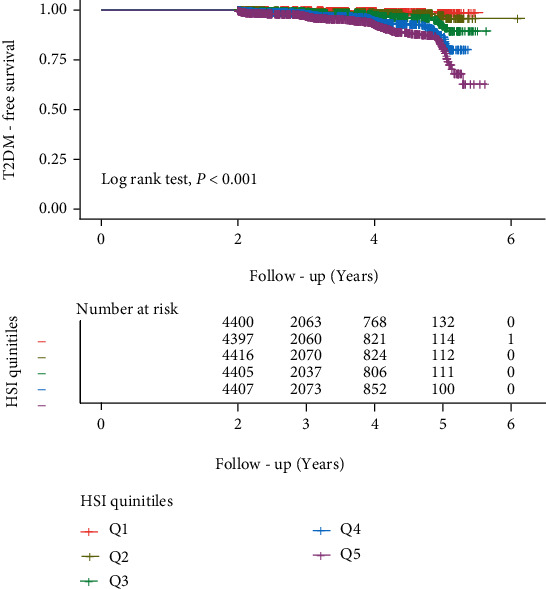
Kaplan-Meier analysis of incident T2DM according to the HSI quintiles. The vertical axis is the diabetes-free survival rate, and the horizontal axis is the follow-up time (years).

During a median follow-up period of 2.97 years (IQR, 2.17–3.88), 433 (1.97%) participants had new-onset T2DM. The cumulative prevalence of T2DM was 0.30% (13/4400) in the Q1 group, 0.75% (33/4397) in the Q2 group, 1.45% (64/4416) in the Q3 group, 2.91% (128/4405) in the Q4 group, and 4.42% (195/4407) in the Q5 group.  Figure 2 demonstrates the results of Kaplan-Meier analysis based on HSI quintiles, with a progressive increase in the cumulative prevalence of T2DM with increasing HSI (log-rank test *P* < 0.001).

### 3.3. The Relationship of HSI with the Risk of T2DM

**Figure 3:**
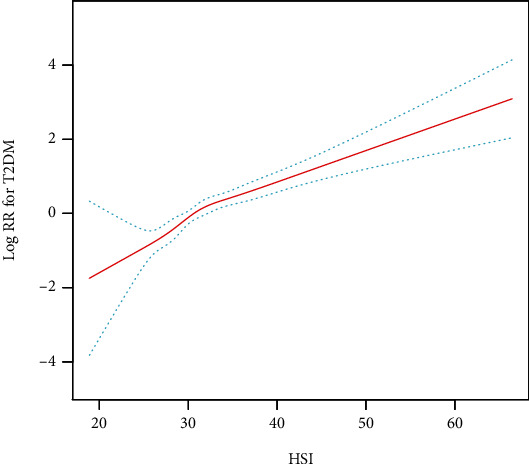
The association between HSI and the risk of T2DM. ^∗^The spline was adjusted for all noncollinear variables.

The smoothing curve fit plots revealed a positive correlation between HSI and the risk of T2DM (Figure 3 and Supplement Figure 1). Overall, there was a significant positive association between HSI and the risk of new-onset T2DM in the multivariate regression model (Table 2). In model IV, the risk of T2DM increased by 62% for every 1 SD increase in HSI (HR: 1.62, 95% CI: 1.41–1.89). When HSI was assessed as quintiles, compared to those in quintile 1, the adjusted HRs (95% CI) for new-onset T2DM in quintile 2, quintile 3, quintile 4, and quintile 5 were 1.66 (0.85-3.22), 1.82 (0.98-3.46), 3.19 (1.64-5.92), and 3.48 (1.85-7.16; *P* for trend, <0.001), respectively. The kernel outcomes of the complete data analysis were in agreement with the original data (Supplementary Table 4). Further, to validate the robustness of the primary analysis outcomes, investigators performed sensitivity analyses after excluding current smokers and drinkers, respectively, and these results demonstrated equivalent independent correlations (Supplementary Tables 5 and 6).

### 3.4. Independent Association of HSI with the Risk of T2DM in Different Subgroups

**Figure 4:**
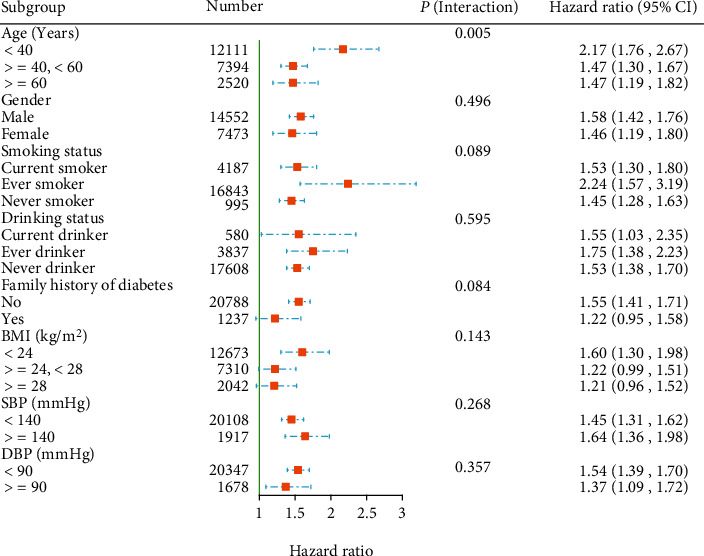
Subgroup analysis of associations between HSI (per 1 SD increment) and risk of T2DM. ^∗^Adjusted for all noncollinear variables, if not be stratified.

The results of subgroup analysis indicated that age played an interactive role between HSI and the risk of T2DM (*P* for interaction, 0.005). Higher HSI levels were related to a higher risk of T2DM (HR: 2.17, 95% CI: 1.76-2.67) in those aged < 40 years. Other variables did not substantially modify the association between HSI levels and risk of T2DM (Figure 4).

### 3.5. The Discriminative Power of HSI for T2DM

**Figure 5:**
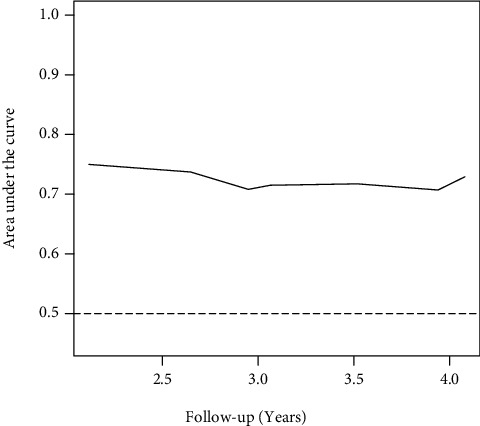
Time-dependent receiver operating characteristic curve.

We evaluated the discriminative power of HSI for T2DM at different time points (Figure 5). The AUCs were 0.711 at 3 years and 0.717 at 4 years, which indicated helpful discrimination for T2DM.

### 3.6. The Incremental Impact of HSI on the Predictive Value for New-Onset T2DM

We further assessed the predictive ability of HSI beyond established risk factors for new-onset T2DM (Table 3). First, the Hosmer-Lemeshow test revealed that the model calibration was adequate with the addition of HSI to established risk factors (*P* > 0.05). Second, Table 3 demonstrated that the addition of HSI significantly improved the reclassification and discrimination of established risk factors with an IDI of 0.027 and an NRI of 0.348 (both *P* < 0.001). Furthermore, the *C*-statistics of established risk factors [0.791 (0.769-0.812)] changed after the addition of the HSI [0.846 (0.829-0.863), *P* < 0.001].

## 4. Discussion

T2DM is a substantial public health and economic problem worldwide and is common in the general population [32–34]. Thus, it is crucial that individuals at high risk for T2DM are identified, which may contribute to avoiding an unprecedented increase in the incidence of the disease [8, 35]. Furthermore, the results of the subgroup analysis demonstrated that a stronger association between HSI and the risk of T2DM was observed in participants aged < 40 years. This study provides the first evidence of an independent association between HSI and new-onset T2DM, and the addition of HSI to the baseline model significantly enhanced the performance of predicting the risk of T2DM.

Substantial research has confirmed that hepatic fat accumulation is an independent risk factor for the risk of T2DM [15, 36]. In a longitudinal study of 129 Swedish adults with biopsy-proven NAFLD and elevated serum transaminase levels, the prevalence of T2DM and impaired glucose tolerance increased from 8.5% at baseline to 80% at the end of 14 years [37]. Similarly, in a Korean retrospective cohort study, a total of 13218 participants without T2DM at baseline were enrolled and followed for 5 years [38]. In this study, those patients with NAFLD who progressed to a more severe stage had a significantly increased risk of new-onset T2DM compared to subjects with NAFLD in remission over the same period (OR: 2.49, 95% CI: 1.49-4.14) [38]. In addition, the results of a prospective cohort study conducted in Spain demonstrated that hepatic steatosis was strongly associated with the risk of new-onset T2DM during follow-up. And this association was independent of possible confounding factors such as lifestyle, family history of diabetes, education level, lipid levels, hypertension, and transaminase levels [39]. Likewise, similar conclusions were reached in an open-label, cluster-randomized trial (DiRECT) [40]. In a 5-year observational cohort study, Busquets-Cortés et al. [41] included a total of 16,648 adults with prediabetes. This study further demonstrated that regular physical activity and a healthy diet can help reverse prediabetes by improving the degree of hepatic steatosis.

The HSI, recently developed by Lee et al., can be used as a simple tool to screen for hepatic steatosis [23]. Liver biopsy has long been considered the “gold standard” for the diagnosis of hepatic steatosis [42]. However, due to its invasive nature and resulting complications, it has not been widely used in clinical practice. Radiological diagnosis using ultrasonography, computed tomography, and magnetic resonance imaging has been demonstrated to accurately assess the extent of hepatic steatosis [43–45]. However, there are limitations to this noninvasive examination, including the high cost of evaluation and the specialized equipment required. Therefore, the great advantages of HSI are its simplicity, accuracy, and accessibility. A survey by Wang et al. revealed that HSI was significantly and positively correlated with hepatic insulin resistance and abnormal lipid metabolism [17]. Additionally, HSI was positively correlated with fasting blood insulin and C-peptide, TG, TC, and LDL-C levels, but negatively correlated with HDL-C. This was similar to the findings of Kitade et al. [25]. In addition, Kitade et al. revealed a positive correlation between HSI and insulin resistance and *β*-cell function in a nondiabetic population [25]. Tripolino et al. and Sviklāne et al. both found a strong correlation between HSI and risk indicators of lipid metabolism [19, 46]. The results of all these studies indicate that HSI is strongly associated with insulin resistance and lipid metabolism disorders and that HSI has better predictive value as a predictor of T2DM. Therefore, this study used a large sample size and longitudinal design to confirm a causal relationship between HSI and T2DM in Chinese adults, independent of traditional risk factors. The reasons for this strange finding are unclear, but it may be essential to note the following two points. First, with rapid economic development, young people will inevitably reduce their need for physical activity and are more prone to various metabolic diseases [47, 48]. Second, in modern society, young people have developed increasingly unhealthy lifestyle habits, leading to the premature development of multiple metabolic diseases [49, 50].

The exact mechanism of the association between HSI and the risk of T2DM remains unclear. However, there are several possible explanations. Insulin resistance is linked to hepatic steatosis [51, 52]. In animal models of hepatic steatosis, the ability of insulin to inhibit hepatic gluconeogenesis is diminished even when muscle insulin resistance is not significantly altered [53]. Hepatic steatosis or hepatic fat infiltration may induce hepatic insulin resistance through activation of JNK1 and PKC-epsilon, which may interfere with tyrosine phosphorylation of IRS-1 and IRS-2. This further contributes to the impaired ability of insulin to activate glycogen synthesis and inhibit gluconeogenesis [54]. In addition, another possible mechanism may involve inflammatory effects in the liver that impair insulin signaling, resulting in the inability to inhibit glucose production and the eventual development of hyperglycemia [55, 56].

The greatest advantage of the study is that it is from a multicenter, large-scale cohort study in China. Therefore, there was a sufficient sample size available for analysis to confirm the robustness and reliability of the outcomes. Secondly, the independent relationship between HSI and T2DM was confirmed after adjustment for a series of conventional risk factors. Further, *C*-statistic, IDI, and NRI analyses validated the incremental predictive value of HSI over and above the established risk factors. Finally, a variety of sensitivity analyses were conducted in the current research to improve the rigor of the findings. With these reliable statistical analyses, it can be concluded that the conclusions of this study are more reliable, and the results can be applicable to most Chinese populations.

The strengths of this study are evident, but several limitations should also be considered when making cautious interpretations. First, the 2-hour oral glucose tolerance test was not used in this study to diagnose T2DM, so this may have resulted in missing cases of new-onset T2DM. Second, patient follow-up was relatively short, and the primary effect of shorter follow-up was a lower rate of endpoint events. Finally, the findings of this study are currently applicable mainly to the Chinese population. Therefore, the applicability of the study results to other populations or ethnicities needs to be further investigated.

## 5. Conclusions

In general, our findings demonstrate that an elevated HSI is significantly associated with a greater risk of T2DM. HSI may be an accessible and supplementary monitoring method in the management of T2DM risk stratification in the Chinese population.

## Figures and Tables

**Table 1:** Baseline characteristics of the overall participants stratified by HSI quintiles.

HSI	*Q*1 (18.87-27.23)	*Q*2 (27.24-29.79)	*Q*3 (29.80-32.42)	*Q*4 (32.43-35.97)	*Q*5 (35.98-66.44)	*P* value
No. of participants	4400	4397	4416	4405	4407	
Age (years)	37.89 ± 12.14	41.27 ± 12.51	43.12 ± 12.52	44.20 ± 12.67	41.20 ± 10.89	<0.001
Gender, *n* (%)						<0.001
Male	2280 (51.82%)	2429 (55.24%)	2842 (64.36%)	3263 (74.07%)	3738 (84.82%)	
Female	2120 (48.18%)	1968 (44.76%)	1574 (35.64%)	1142 (25.93%)	669 (15.18%)	
Height (cm)	166.16 ± 8.08	166.25 ± 8.25	167.08 ± 8.27	168.19 ± 8.22	170.05 ± 7.89	<0.001
Weight (kg)	54.19 ± 7.17	60.38 ± 7.82	65.56 ± 8.19	71.07 ± 8.45	79.53 ± 10.39	<0.001
BMI (kg/m^2^)	19.56 ± 1.53	21.77 ± 1.53	23.42 ± 1.67	25.08 ± 1.87	27.46 ± 2.69	<0.001
SBP (mmHg)	113.05 ± 14.16	115.83 ± 14.99	118.54 ± 14.96	122.08 ± 15.17	125.54 ± 14.80	<0.001
DBP (mmHg)	70.59 ± 9.31	72.15 ± 9.70	74.00 ± 10.02	76.45 ± 10.08	79.21 ± 10.26	<0.001
FPG (mmol/L)	4.81 ± 0.61	4.89 ± 0.61	4.98 ± 0.64	5.05 ± 0.64	5.12 ± 0.65	<0.001
TC (mmol/L)	4.41 ± 0.83	4.61 ± 0.85	4.74 ± 0.88	4.87 ± 0.89	5.00 ± 0.91	<0.001
TG (mmol/L)	0.89 ± 0.52	1.10 ± 0.73	1.36 ± 1.01	1.71 ± 1.27	2.04 ± 1.31	<0.001
HDL-C (mmol/L)	1.46 ± 0.29	1.42 ± 0.29	1.36 ± 0.34	1.30 ± 0.28	1.23 ± 0.28	<0.001
LDL-C (mmol/L)	2.51 ± 0.62	2.65 ± 0.66	2.74 ± 0.68	2.82 ± 0.69	2.91 ± 0.71	<0.001
ALT (IU/L)	12.00 (10.00-15.10)	15.00 (12.00-19.30)	19.00 (15.00-24.42)	25.00 (19.00-32.30)	40.20 (29.40-59.00)	<0.001
AST (IU/L)	21.00 (18.30-24.80)	21.20 (18.20-25.10)	22.40 (19.00-27.00)	24.00 (20.30-29.00)	28.00 (22.80-36.00)	<0.001
HSI	25.28 ± 1.47	28.53 ± 0.73	31.07 ± 0.76	34.04 ± 1.01	39.87 ± 3.50	<0.001
BUN (mmol/L)	4.48 ± 1.17	4.59 ± 1.18	4.70 ± 1.17	4.86 ± 1.18	4.85 ± 1.14	<0.001
Scr (mmol/L)	69.81 ± 15.04	70.75 ± 15.59	72.96 ± 15.16	75.21 ± 14.94	76.92 ± 13.90	<0.001
Smoking status, *n* (%)						<0.001
Current smoker	584 (13.27%)	639 (14.53%)	770 (17.44%)	952 (21.61%)	1242 (28.18%)	
Ever smoker	129 (2.93%)	156 (3.55%)	188 (4.26%)	240 (5.45%)	282 (6.40%)	
Never smoker	3687 (83.80%)	3602 (81.92%)	3458 (78.31%)	3213 (72.94%)	2883 (65.42%)	
Drinking status, *n* (%)						<0.001
Current drinker	101 (2.30%)	89 (2.02%)	103 (2.33%)	147 (3.34%)	140 (3.18%)	
Ever drinker	519 (11.80%)	608 (13.83%)	784 (17.75%)	900 (20.43%)	1026 (23.28%)	
Never drinker	3780 (85.91%)	3700 (84.15%)	3529 (79.91%)	3358 (76.23%)	3241 (73.54%)	
Family history of diabetes, *n* (%)						<0.001
No	4246 (96.50%)	4174 (94.93%)	4134 (93.61%)	4138 (93.94%)	4096 (92.94%)	
Yes	154 (3.50%)	223 (5.07%)	282 (6.39%)	267 (6.06%)	311 (7.06%)	

The variables are presented as mean ± SD or median (quartile 1-quartile 3) or *n* (%).

**Table 2:** Association between HSI and risk of T2DM in different models.

	Crude model	Model I	Model II	Model III	Model IV
	HR (95% CI)	HR (95% CI)	HR (95% CI)	HR (95% CI)	HR (95% CI)
Continuous					
HSI (per SD increase)	1.86 (1.73, 2.00)	2.09 (1.93, 2.27)	1.63 (1.42, 1.87)	1.62 (1.40, 1.87)	1.62 (1.41, 1.89)
Categorical					
HSI (quintile)					
*Q*1	Ref	Ref	Ref	Ref	Ref
*Q*2	2.54 (1.34, 4.83)	2.04 (1.08, 3.88)	1.68 (0.88, 3.23)	1.63 (0.85, 3.13)	1.66 (0.85, 3.22)
*Q*3	4.90 (2.70, 8.90)	3.47 (1.91, 6.29)	1.84 (0.98, 3.44)	1.75 (0.93, 3.29)	1.82 (0.98, 3.46)
*Q*4	9.98 (5.64, 17.66)	6.78 (3.83, 12.00)	3.01 (1.60, 5.66)	2.99 (1.58, 5.64)	3.19 (1.64, 5.92)
*Q*5	15.24 (8.69, 26.72)	13.05 (7.44, 22.90)	3.46 (1.75, 6.83)	3.24 (1.62, 6.47)	3.48 (1.85, 7.16)
*P* for trend	< 0.001	< 0.001	< 0.001	< 0.001	< 0.001

Crude model adjusted for none. Model I adjusted for gender and age. Model II adjusted for age, SBP, DBP, FPG, TG, and BMI. Model III adjusted for age, gender, BMI, SBP, DBP, FPG, TG, LDL-C, AST, BUN, smoking status, drinking status, and family history of diabetes. Model IV adjusted for age, gender, BMI, SBP, DBP, FPG, TG, HDL-C, LDL-C, AST, BUN, Scr, smoking status, drinking status, and family history of diabetes. Abbreviations: Ref: reference; CI: confidence interval; HR: hazard ratios.

**Table 3:** Discrimination of predictive model for risk of T2DM using *C*-statistics, NRI, and IDI.

	*C*-statistic	*P* value	NRI (95% CI)	*P* value	IDI (95% CI)	*P* value
Established risk factors	0.791 (0.769-0.812)	Ref.		Ref.		Ref.
Established risk factors+HSI	0.846 (0.829-0.863)	<0.001	0.348 (0.284-0.410)	<0.001	0.027 (0.018-0.038)	<0.001

Established risk factors included age, gender, family history of diabetes, smoking status, and drinking status. Abbreviations: Ref: reference; IDI: integrated discrimination improvement; NRI: net reclassification improvement.

## Data Availability

Raw dataset is stored in Dryad (10.5061/dryad.ft8750v).

## Supplementary Materials

Supplementary Materials Table 1: the description of missing data. Table 2: sensitivity comparative analysis between preimputation and postimputation. Table 3: collinearity diagnostics steps. Table 4: results of multivariate Cox regression among original data and postimputation data. Table 5: HRs and 95% CI for risk of type 2 diabetes mellitus excluding current smoker. Table 6: HRs and 95% CI for risk of type 2 diabetes mellitus excluding current drinker. Figure 1: the association between HSI and the risk of T2DM in postimputation data.
