# Supplementary material for: Hepatic Steatosis Index and the Risk of Type 2 Diabetes Mellitus in China: Insights from a General Population-Based Cohort Study
Source: Dis Markers. 2022 Aug 3;2022:3150380. doi: 10.1155/2022/3150380 (PMC9365599; doi:10.1155/2022/3150380)
Supplement: Supplementary Materials — Table 1: the description of missing data. Table 2: sensitivity comparative analysis between preimputation and postimputation. Table 3: collinearity diagnostics steps. Table 4: results of multivariate Cox regression among original data and postimputation data. Table 5: HRs and 95% CI for risk of type 2 diabetes mellitus excluding current smoker. Table 6: HRs and 95% CI for risk of type 2 diabetes mellitus excluding current drinker. Figure 1: the association between HSI and the risk of T2DM in postimputation data. [file 3150380.f1.docx]

**Supplemental Material**

**Supplementary Methods:**

**The Details of Statistical Analysis**

For population characteristics based on baseline HSI quintiles, descriptive data are presented as mean [standard deviation (SD)] or median with interquartile range (IQR) or proportions, as appropriate.

Cox regression models were performed to estimate the relationship between HSI and the risk of T2DM, and hazard ratios (HR) and 95% confidence intervals (CI) were calculated. The collinearity among the covariates was assessed by calculating the VIF of the covariates before building the model (Supplementary Table 3) [30]. Kaplan-Meier survival curves were then employed to show the risk of T2DM for each HSI quintile, and compliance with the proportional risk assumptions used to build the Cox model was ascertained by looking at the Kaplan-Meier curves corresponding to the HSI quintile. Following the above premise, we implemented a model adjustment strategy concerning the statement of the Strengthening the Reporting of Observational Studies in Epidemiology (STROBE) [31]. HSI was presented as a continuous variable (per SD increase) and a categorical variable (quintiles) and was placed into different models. In addition to the crude model, we adjusted for various confounders, and four regression models were built. Model I adjusted only for basic demographic data (gender and age). Model II adjusted for variables that had >10% effect on the association between HSI and the risk of T2DM (age, SBP, DBP, FPG, TG, and BMI). Model III adjusted for variables with P<0.1 in the simple Cox regression model + model II (age, gender, BMI, SBP, DBP, FPG, TG, LDL-C, AST, BUN, smoking status, drinking status, and family history of diabetes). Model IV adjusted for all non-collinear variables (age, gender, BMI, SBP, DBP, FPG, TG, HDL-C, LDL-C, AST, BUN, Scr, smoking status, drinking status, and family history of diabetes). To validate the robustness of the results derived from the primary analyses, sensitivity analyses were conducted, excluding participants who were current smokers and drinkers.

Furthermore, we performed a stratified analysis with interaction tests. Stratification of the study included family history of diabetes (no and yes), age (≥60, ≥40 and <60, and <40 years), sex (male and female), BMI (≥28, ≥24 and <28, and <24 kg/m^2^), SBP (≥140 and <140 mmHg), DBP (≥90 and <90 mmHg), smoking status (never, past, and current), and alcohol consumption status (never, past, and current).

Lastly, the time-dependent receiver operating curve (ROC) and the area under the ROC (AUC) were used to evaluate the discriminatory ability of the HSI for T2DM at different time points. The Hosmer-Lemeshow χ^2^ statistic was employed to assess the calibration of models with HSI. Additionally, we used the C-statistic, integrated discrimination improvement (IDI), and net reclassification index (NRI) to assess the incremental predictive value of the HSI over and above the established risk factors [32].

Statistical analyses were performed using R software, version 4.0.1.

Supplement Table 1: The description of missing data.

| Variables | Non-missing | Missing |
| --- | --- | --- |
| Age | 22025 | 0 |
| Gender | 22025 | 0 |
| Height | 22024 | 0 |
| Weight | 22025 | 0 |
| BMI | 22025 | 0 |
| SBP | 22022 | 3 |
| DBP | 22022 | 3 |
| FPG | 22025 | 0 |
| TC | 21855 | 170 |
| TG | 21855 | 170 |
| HDL-C | 12815 | 9210 |
| LDL-C | 13519 | 8506 |
| ALT | 22025 | 0 |
| AST | 22025 | 0 |
| HSI | 22025 | 0 |
| BUN | 20060 | 1965 |
| Scr | 21422 | 603 |
| Smoking status | 22025 | 0 |
| Drinking status | 22025 | 0 |
| Family history of diabetes | 22025 | 0 |

Abbreviations as in Table 1.

Supplementary Table 2: Sensitivity comparative analysis between pre-imputation and post-imputation.

|  | Original data | Post-imputation 1 | Post-imputation 2 | Post-imputation 3 | Post-imputation 4 | Post-imputation 5 | P-value |
| --- | --- | --- | --- | --- | --- | --- | --- |
| No. of subjects | 22025 | 22025 | 22025 | 22025 | 22025 | 22025 |  |
| Age (years) | 41.54 ± 12.35 | 41.54 ± 12.35 | 41.54 ± 12.35 | 41.54 ± 12.35 | 41.54 ± 12.35 | 41.54 ± 12.35 | 1.000 |
| Gender, n (%) |  |  |  |  |  |  | 1.000 |
| Male | 14552 (66.07%) | 14552 (66.07%) | 14552 (66.07%) | 14552 (66.07%) | 14552 (66.07%) | 14552 (66.07%) |  |
| Female | 7473 (33.93%) | 7473 (33.93%) | 7473 (33.93%) | 7473 (33.93%) | 7473 (33.93%) | 7473 (33.93%) |  |
| Height (cm) | 167.55 ± 8.27 | 167.55 ± 8.27 | 167.55 ± 8.27 | 167.55 ± 8.27 | 167.55 ± 8.27 | 167.55 ± 8.27 | 1.000 |
| Weight (kg) | 66.15 ± 12.16 | 66.15 ± 12.16 | 66.15 ± 12.16 | 66.15 ± 12.16 | 66.15 ± 12.16 | 66.15 ± 12.16 | 1.000 |
| BMI (kg/m^2^) | 23.46 ± 3.31 | 23.46 ± 3.31 | 23.46 ± 3.31 | 23.46 ± 3.31 | 23.46 ± 3.31 | 23.46 ± 3.31 | 1.000 |
| SBP (mmHg) | 119.01 ± 15.46 | 119.01 ± 15.46 | 119.01 ± 15.46 | 119.01 ± 15.46 | 119.01 ± 15.46 | 119.01 ± 15.46 | 1.000 |
| DBP (mmHg) | 74.48 ± 10.34 | 74.48 ± 10.34 | 74.48 ± 10.34 | 74.48 ± 10.35 | 74.48 ± 10.35 | 74.48 ± 10.34 | 1.000 |
| FPG (mmol/L) | 4.97 ± 0.64 | 4.97 ± 0.64 | 4.97 ± 0.64 | 4.97 ± 0.64 | 4.97 ± 0.64 | 4.97 ± 0.64 | 1.000 |
| TC (mmol/L) | 4.72 ± 0.89 | 4.73 ± 0.89 | 4.73 ± 0.90 | 4.73 ± 0.89 | 4.73 ± 0.90 | 4.73 ± 0.90 | 0.999 |
| TG (mmol/L) | 1.42 ± 1.10 | 1.41 ± 1.10 | 1.42 ± 1.10 | 1.42 ± 1.10 | 1.42 ± 1.10 | 1.42 ± 1.10 | 1.000 |
| HDL-C (mmol/L) | 1.35 ± 0.31 | 1.35 ± 0.31 | 1.34 ± 0.31 | 1.35 ± 0.31 | 1.34 ± 0.31 | 1.35 ± 0.31 | 0.873 |
| LDL-C (mmol/L) | 2.73 ± 0.69 | 2.72 ± 0.69 | 2.72 ± 0.69 | 2.71 ± 0.69 | 2.71 ± 0.69 | 2.71 ± 0.69 | 0.095 |
| ALT (IU/L) | 25.51 ± 22.45 | 25.51 ± 22.45 | 25.51 ± 22.45 | 25.51 ± 22.45 | 25.51 ± 22.45 | 25.51 ± 22.45 | 1.000 |
| AST (IU/L) | 25.15 ± 10.68 | 25.15 ± 10.68 | 25.15 ± 10.68 | 25.15 ± 10.68 | 25.15 ± 10.68 | 25.15 ± 10.68 | 1.000 |
| HSI | 31.76 ± 5.30 | 31.76 ± 5.30 | 31.76 ± 5.30 | 31.76 ± 5.30 | 31.76 ± 5.30 | 31.76 ± 5.30 | 1.000 |
| BUN (mmol/L) | 4.70 ± 1.18 | 4.72 ± 1.18 | 4.72 ± 1.18 | 4.72 ± 1.19 | 4.72 ± 1.18 | 4.71 ± 1.18 | 0.458 |
| Scr (mmol/L) | 73.15 ± 15.17 | 73.09 ± 15.19 | 73.10 ± 15.18 | 73.07 ± 15.19 | 73.08 ± 15.20 | 73.09 ± 15.17 | 0.997 |
| Smoking status, n (%) |  |  |  |  |  |  | 1.000 |
| Current smoker | 4187 (19.01%) | 4187 (19.01%) | 4187 (19.01%) | 4187 (19.01%) | 4187 (19.01%) | 4187 (19.01%) |  |
| Ever smoker | 995 (4.52%) | 995 (4.52%) | 995 (4.52%) | 995 (4.52%) | 995 (4.52%) | 995 (4.52%) |  |
| Never smoker | 16843 (76.47%) | 16843 (76.47%) | 16843 (76.47%) | 16843 (76.47%) | 16843 (76.47%) | 16843 (76.47%) |  |
| Drinking status, n (%) |  |  |  |  |  |  | 1.000 |
| Current drinker | 580 (2.63%) | 580 (2.63%) | 580 (2.63%) | 580 (2.63%) | 580 (2.63%) | 580 (2.63%) |  |
| Ever drinker | 3837 (17.42%) | 3837 (17.42%) | 3837 (17.42%) | 3837 (17.42%) | 3837 (17.42%) | 3837 (17.42%) |  |
| Never drinker | 17608 (79.95%) | 17608 (79.95%) | 17608 (79.95%) | 17608 (79.95%) | 17608 (79.95%) | 17608 (79.95%) |  |
| Family history of diabetes, n (%) |  |  |  |  |  |  | 1.000 |
| No | 20788 (94.38%) | 20788 (94.38%) | 20788 (94.38%) | 20788 (94.38%) | 20788 (94.38%) | 20788 (94.38%) |  |
| Yes | 1237 (5.62%) | 1237 (5.62%) | 1237 (5.62%) | 1237 (5.62%) | 1237 (5.62%) | 1237 (5.62%) |  |

Values are n(%) or mean ± SD. Abbreviations as in Table 1.

Supplementary Table 3: Collinearity diagnostics steps.

|  | Step 1 | Step 2 | Step 3 | Step 4 |
| --- | --- | --- | --- | --- |
| HSI | 12.9 | 12.9 | 4.0 | 4.0 |
| Age | 1.3 | 1.3 | 1.3 | 1.3 |
| Gender | 3.4 | 3.4 | 3.2 | 3.2 |
| Height | 52.4 | NA | NA | NA |
| Weight | 173.2 | NA | NA | NA |
| BMI | 109.9 | 7.7 | 4.0 | 4.0 |
| SBP | 2.0 | 2.0 | 2.0 | 2.0 |
| DBP | 2.0 | 2.0 | 2.0 | 2.0 |
| FPG | 1.1 | 1.1 | 1.1 | 1.1 |
| TC | 6.2 | 6.2 | 6.2 | NA |
| TG | 1.7 | 1.7 | 1.7 | 1.3 |
| HDL-C | 1.4 | 1.4 | 1.4 | 1.2 |
| LDL-C | 5.2 | 5.2 | 5.2 | 1.1 |
| ALT | 11.1 | 11.1 | NA | NA |
| AST | 5.7 | 5.7 | 1.2 | 1.2 |
| BUN | 1.2 | 1.2 | 1.2 | 1.2 |
| Scr | 2.1 | 2.1 | 2.1 | 2.1 |
| Smoking status | 1.3 | 1.3 | 1.3 | 1.3 |
| Drinking status | 1.2 | 1.2 | 1.2 | 1.2 |
| Family history of diabetes | 1.0 | 1.0 | 1.0 | 1.0 |

VIF: variance inflation factors. Other abbreviations as in Table 1.

VIF = 1/(1-R^2^). VIF step-by-step screening method: Calculate the VIF of each variable. If the maximum VIF value ≥5, remove the variable with the maximum VIF value

Supplementary Table 4: Results of multivariate Cox regression among original data and post-imputation data.

|  | Original data | Post-imputation 1 | Post-imputation 2 | Post-imputation 3 | Post-imputation 4 | Post-imputation 5 | Total |
| --- | --- | --- | --- | --- | --- | --- | --- |
|  | HR (95%CI) | HR (95%CI) | HR (95%CI) | HR (95%CI) | HR (95%CI) | HR (95%CI) | HR (95%CI) |
| Crude Model |  |  |  |  |  |  |  |
| HSI (per SD increase) | 1.86 (1.73, 2.00) | 1.86 (1.73, 2.00) | 1.86 (1.73, 2.00) | 1.86 (1.73, 2.00) | 1.86 (1.73, 2.00) | 1.86 (1.73, 2.00) | 1.86 (1.81, 1.92) |
| HSI (quintile) |  |  |  |  |  |  |  |
| Q1 | Ref | Ref | Ref | Ref | Ref | Ref | Ref |
| Q2 | 2.54 (1.34, 4.83) | 2.54 (1.34, 4.83) | 2.54 (1.34, 4.83) | 2.54 (1.34, 4.83) | 2.54 (1.34, 4.83) | 2.54 (1.34, 4.83) | 2.54 (1.96, 3.31) |
| Q3 | 4.90 (2.70, 8.90) | 4.90 (2.70, 8.90) | 4.90 (2.70, 8.90) | 4.90 (2.70, 8.90) | 4.90 (2.70, 8.90) | 4.90 (2.70, 8.90) | 4.90 (3.84, 6.25) |
| Q4 | 9.98 (5.64, 17.66) | 9.98 (5.64, 17.66) | 9.98 (5.64, 17.66) | 9.98 (5.64, 17.66) | 9.98 (5.64, 17.66) | 9.98 (5.64, 17.66) | 9.98 (7.91, 12.60) |
| Q5 | 15.24 (8.69, 26.72) | 15.24 (8.69, 26.72) | 15.24 (8.69, 26.72) | 15.24 (8.69, 26.72) | 15.24 (8.69, 26.72) | 15.24 (8.69, 26.72) | 15.24 (12.12, 19.17) |
| P for trend | < 0.001 | < 0.001 | < 0.001 | < 0.001 | < 0.001 | < 0.001 | < 0.001 |
| Model I |  |  |  |  |  |  |  |
| HSI (per SD increase) | 2.09 (1.93, 2.27) | 2.09 (1.93, 2.27) | 2.09 (1.93, 2.27) | 2.09 (1.93, 2.27) | 2.09 (1.93, 2.27) | 2.09 (1.93, 2.27) | 2.09 (2.02, 2.16) |
| HSI (quintile) |  |  |  |  |  |  |  |
| Q1 | Ref | Ref | Ref | Ref | Ref | Ref | Ref |
| Q2 | 2.04 (1.08, 3.88) | 2.04 (1.08, 3.88) | 2.04 (1.08, 3.88) | 2.04 (1.08, 3.88) | 2.04 (1.08, 3.88) | 2.04 (1.08, 3.88) | 2.04 (1.57, 2.65) |
| Q3 | 3.47 (1.91, 6.29) | 3.47 (1.91, 6.29) | 3.47 (1.91, 6.29) | 3.47 (1.91, 6.29) | 3.47 (1.91, 6.29) | 3.47 (1.91, 6.29) | 3.47 (2.72, 4.42) |
| Q4 | 6.78 (3.83, 12.00) | 6.78 (3.83, 12.00) | 6.78 (3.83, 12.00) | 6.78 (3.83, 12.00) | 6.78 (3.83, 12.00) | 6.78 (3.83, 12.00) | 6.78 (5.37, 8.56) |
| Q5 | 13.05 (7.44, 22.90) | 13.05 (7.44, 22.90) | 13.05 (7.44, 22.90) | 13.05 (7.44, 22.90) | 13.05 (7.44, 22.90) | 13.05 (7.44, 22.90) | 13.05 (10.37, 16.42) |
| P for trend | < 0.001 | < 0.001 | < 0.001 | < 0.001 | < 0.001 | < 0.001 | < 0.001 |
| Model II |  |  |  |  |  |  |  |
| HSI (per SD increase) | 1.63 (1.42, 1.87) | 1.62 (1.42, 1.86) | 1.62 (1.42, 1.86) | 1.62 (1.42, 1.86) | 1.62 (1.42, 1.86) | 1.62 (1.41, 1.86) | 1.63 (1.54, 1.72) |
| HSI (quintile) |  |  |  |  |  |  |  |
| Q1 | Ref | Ref | Ref | Ref | Ref | Ref | Ref |
| Q2 | 1.68 (0.88, 3.23) | 1.68 (0.88, 3.21) | 1.68 (0.88, 3.22) | 1.68 (0.88, 3.22) | 1.68 (0.88, 3.21) | 1.68 (0.87, 3.21) | 1.68 (1.29, 2.19) |
| Q3 | 1.84 (0.98, 3.44) | 1.84 (0.98, 3.43) | 1.84 (0.98, 3.44) | 1.84 (0.98, 3.43) | 1.84 (0.98, 3.43) | 1.84 (0.98, 3.43) | 1.84 (1.42, 2.37) |
| Q4 | 3.01 (1.60, 5.66) | 3.05 (1.63, 5.72) | 3.06 (1.63, 5.74) | 3.05 (1.63, 5.72) | 3.05 (1.63, 5.72) | 3.05 (1.63, 5.73) | 3.05 (2.36, 3.94) |
| Q5 | 3.46 (1.75, 6.83) | 3.49 (1.77, 6.87) | 3.50 (1.78, 6.89) | 3.48 (1.77, 6.86) | 3.49 (1.77, 6.87) | 3.49 (1.77, 6.87) | 3.48 (2.64, 4.59) |
| P for trend | < 0.001 | < 0.001 | < 0.001 | < 0.001 | < 0.001 | < 0.001 | < 0.001 |
| Model III |  |  |  |  |  |  |  |
| HSI (per SD increase) | 1.62 (1.40, 1.87) | 1.63 (1.41, 1.89) | 1.62 (1.40, 1.87) | 1.63 (1.41, 1.89) | 1.62 (1.40, 1.88) | 1.62 (1.40, 1.88) | 1.63 (1.52, 1.74) |
| HSI (quintile) |  |  |  |  |  |  |  |
| Q1 | Ref | Ref | Ref | Ref | Ref | Ref | Ref |
| Q2 | 1.63 (0.85, 3.13) | 1.63 (0.85, 3.13) | 1.64 (0.85, 3.14) | 1.63 (0.85, 3.12) | 1.61 (0.84, 3.08) | 1.62 (0.85, 3.12) | 1.62 (1.21, 2.18) |
| Q3 | 1.75 (0.93, 3.29) | 1.75 (0.93, 3.28) | 1.75 (0.93, 3.29) | 1.75 (0.93, 3.29) | 1.72 (0.91, 3.22) | 1.74 (0.92, 3.26) | 1.74 (1.31, 2.31) |
| Q4 | 2.99 (1.58, 5.64) | 3.00 (1.59, 5.66) | 3.03 (1.61, 5.72) | 3.00 (1.59, 5.67) | 2.95 (1.56, 5.56) | 2.99 (1.58, 5.64) | 2.99 (2.25, 3.98) |
| Q5 | 3.24 (1.62, 6.47) | 3.30 (1.65, 6.60) | 3.38 (1.69, 6.75) | 3.34 (1.67, 6.67) | 3.24 (1.62, 6.47) | 3.28 (1.64, 6.56) | 3.31 (2.43, 4.51) |
| P for trend | < 0.001 | < 0.001 | < 0.001 | < 0.001 | < 0.001 | < 0.001 | < 0.001 |
| Model IV |  |  |  |  |  |  |  |
| HSI (per SD increase) | 1.62 (1.41, 1.89) | 1.69 (1.46, 1.96) | 1.62 (1.40, 1.87) | 1.67 (1.44, 1.94) | 1.63 (1.41, 1.89) | 1.66 (1.42, 1.93) | 1.65 (1.55, 1.77) |
| HSI (quintile) |  |  |  |  |  |  |  |
| Q1 | Ref | Ref | Ref | Ref | Ref | Ref | Ref |
| Q2 | 1.66 (0.85, 3.22) | 1.73 (0.90, 3.33) | 1.68 (0.88, 3.24) | 1.71 (0.89, 3.29) | 1.65 (0.86, 3.17) | 1.67 (0.87, 3.21) | 1.69 (1.26, 2.26) |
| Q3 | 1.82 (0.98, 3.46) | 1.88 (1.00, 3.55) | 1.82 (0.97, 3.42) | 1.90 (1.01, 3.58) | 1.78 (0.95, 3.35) | 1.81 (0.96, 3.40) | 1.84 (1.38, 2.44) |
| Q4 | 3.19 (1.64, 5.92) | 3.34 (1.76, 6.33) | 3.18 (1.68, 6.02) | 3.39 (1.79, 6.41) | 3.11 (1.64, 5.87) | 3.15 (1.66, 5.95) | 3.23 (2.43, 4.29) |
| Q5 | 3.48 (1.85, 7.16) | 3.69 (1.83, 7.42) | 3.55 (1.77, 7.12) | 3.76 (1.87, 7.57) | 3.39 (1.69, 6.79) | 3.46 (1.72, 6.94) | 3.57 (2.61, 4.87) |
| P for trend | < 0.001 | < 0.001 | < 0.001 | < 0.001 | < 0.001 | < 0.001 | < 0.001 |

Crude model adjusted for none.

Model I adjusted for gender and age.

Model II adjusted for age, SBP, DBP, FPG, TG and BMI.

Model III adjusted for age, gender, BMI, SBP, DBP, FPG, TG, LDL-C, AST, BUN, smoking status, drinking status and family history of diabetes.

Model IV adjusted for age, gender, BMI, SBP, DBP, FPG, TG, HDL-C, LDL-C, AST, BUN, Scr, smoking status, drinking status and family history of diabetes.

Abbreviations: Ref, Reference; CI, confidence interval; HR, hazard ratios. Other abbreviations as in Table 1.

Supplementary Table 5: HRs and 95% CI for risk of type 2 diabetes mellitus excluding current smoker.

|  | Crude Model | Model I | Model II | Model III | Model IV |
| --- | --- | --- | --- | --- | --- |
|  | HR (95%CI) | HR (95%CI) | HR (95%CI) | HR (95%CI) | HR (95%CI) |
| HSI (per SD increase) | 1.89 (1.74, 2.06) | 2.03 (1.85, 2.23) | 1.65 (1.41, 1.95) | 1.70 (1.44, 2.01) | 1.79 (1.51, 2.13) |
| HSI (quintile) |  |  |  |  |  |
| Q1 | Ref | Ref | Ref | Ref | Ref |
| Q2 | 2.49 (1.15, 5.41) | 2.01 (0.93, 4.38) | 1.83 (0.83, 4.01) | 1.85 (0.84, 4.06) | 1.95 (0.88, 4.28) |
| Q3 | 5.51 (2.70, 11.25) | 3.74 (1.83, 7.64) | 2.21 (1.05, 4.68) | 2.20 (1.04, 4.68) | 2.38 (1.12, 5.07) |
| Q4 | 11.16 (5.62, 22.17) | 7.08 (3.56, 14.07) | 3.75 (1.75, 8.01) | 3.87 (1.80, 8.32) | 4.26 (1.98, 9.20) |
| Q5 | 17.52 (8.91, 34.46) | 13.81 (7.02, 27.18) | 4.72 (2.07, 10.75) | 4.96 (2.15, 11.46) | 5.59 (2.40, 13.00) |
| P for trend | < 0.001 | < 0.001 | < 0.001 | < 0.001 | < 0.001 |

Crude model adjusted for none.

Model I adjusted for gender and age.

Model II adjusted for age, SBP, DBP, FPG, TG and BMI.

Model III adjusted for age, gender, BMI, SBP, DBP, FPG, TG, LDL-C, AST, BUN, smoking status, drinking status and family history of diabetes.

Model IV adjusted for age, gender, BMI, SBP, DBP, FPG, TG, HDL-C, LDL-C, AST, BUN, Scr, smoking status, drinking status and family history of diabetes.

Abbreviations: Ref, Reference; CI, confidence interval; HR, hazard ratios. Other abbreviations as in Table 1.

Supplementary Table 6: HRs and 95% CI for risk of type 2 diabetes mellitus excluding current drinker.

|  | Crude Model | Model I | Model II | Model III | Model IV |
| --- | --- | --- | --- | --- | --- |
|  | HR (95%CI) | HR (95%CI) | HR (95%CI) | HR (95%CI) | HR (95%CI) |
| HSI (per SD increase) | 1.88 (1.75, 2.03) | 2.11 (1.94, 2.29) | 1.66 (1.45, 1.91) | 1.68 (1.45, 1.94) | 1.71 (1.47, 1.98) |
| HSI (quintile) |  |  |  |  |  |
| Q1 | Ref | Ref | Ref | Ref | Ref |
| Q2 | 2.39 (1.25, 4.56) | 1.90 (1.00, 3.64) | 1.54 (0.80, 2.97) | 1.47 (0.76, 2.84) | 1.50 (0.78, 2.91) |
| Q3 | 4.69 (2.58, 8.53) | 3.27 (1.80, 5.96) | 1.71 (0.91, 3.22) | 1.58 (0.84, 2.99) | 1.64 (0.87, 3.11) |
| Q4 | 9.56 (5.39, 16.94) | 6.39 (3.60, 11.32) | 2.82 (1.50, 5.33) | 2.70 (1.42, 5.11) | 2.82 (1.48, 5.37) |
| Q5 | 14.61 (8.33, 25.65) | 12.30 (7.00, 21.61) | 3.26 (1.64, 6.48) | 2.95 (1.46, 5.95) | 3.10 (1.53, 6.28) |
| P for trend | < 0.001 | < 0.001 | < 0.001 | < 0.001 | < 0.001 |

Crude model adjusted for none.

Model I adjusted for gender and age.

Model II adjusted for age, SBP, DBP, FPG, TG and BMI.

Model III adjusted for age, gender, BMI, SBP, DBP, FPG, TG, LDL-C, AST, BUN, smoking status, drinking status and family history of diabetes.

Model IV adjusted for age, gender, BMI, SBP, DBP, FPG, TG, HDL-C, LDL-C, AST, BUN, Scr, smoking status, drinking status and family history of diabetes.

Abbreviations: Ref, Reference; CI, confidence interval; HR, hazard ratios. Other abbreviations as in Table 1.


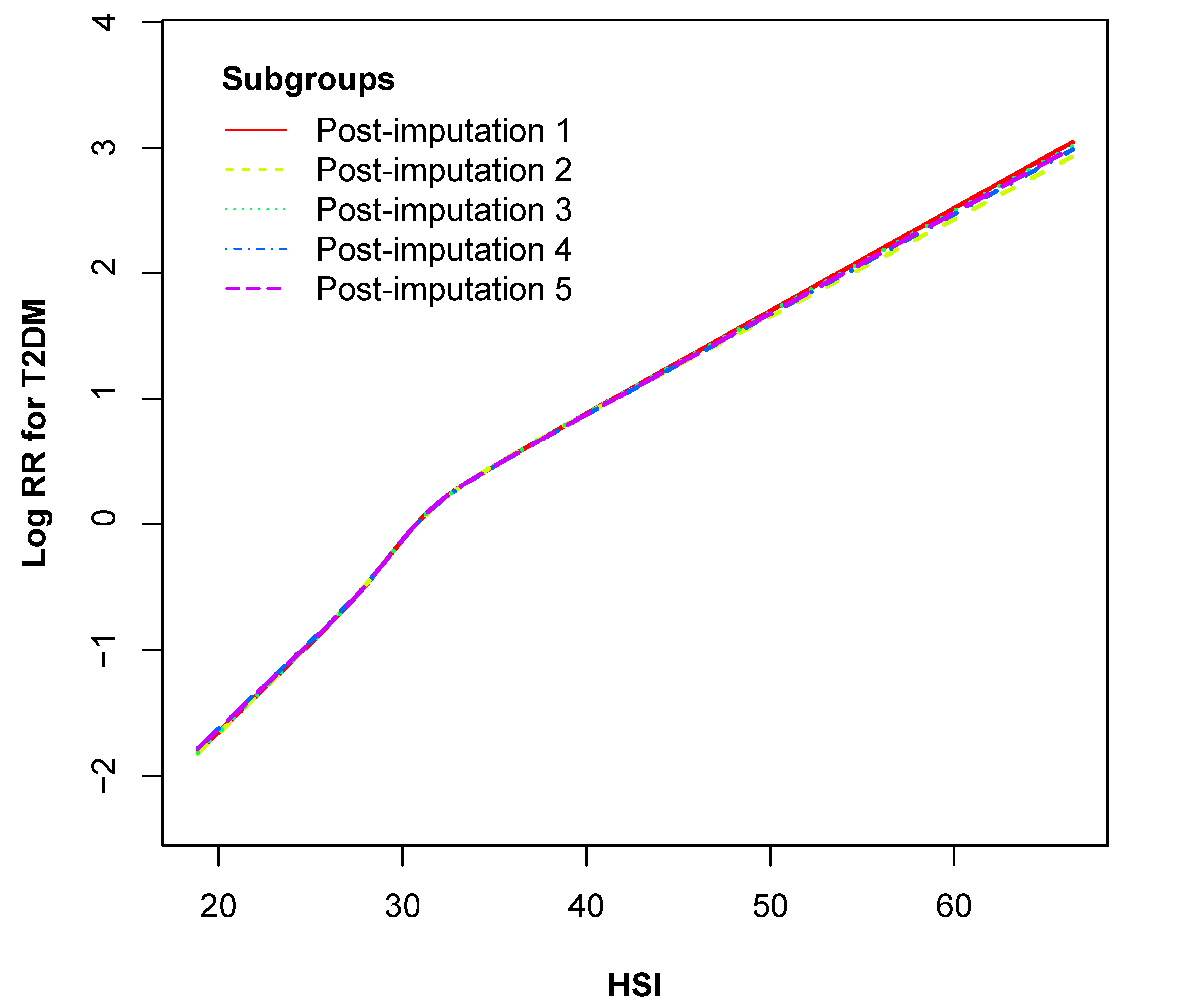


Supplement Figure 1: The association between HSI and the risk of T2DM in post-imputation data.

*The spline was adjusted for all non-collinear variables.
